# Modelling ^85^Kr datasets with python for applications in tracer hydrology and to investigate atmospheric circulation

**DOI:** 10.1016/j.mex.2021.101245

**Published:** 2021-01-23

**Authors:** Arne Kersting, Sofia Brander, Axel Suckow

**Affiliations:** a Institute of Environmental Physics, Heidelberg University, 69120 Heidelberg, Germany; b Bundesamt für Strahlenschutz, Rosastr. 9, 79098 Freiburg, Germany; c CSIRO Land & Water, Gate 5 Waite Road, Urrbrae, SA 5064, Australia

**Keywords:** Krypton-85, Dating water & ice with noble gas radionuclides, Atmospheric circulation

## Abstract

We present a model written in python to evaluate data from comprehensive ^85^Kr collection schemes comprising 11 datasets from different monitoring stations around the globe. The model is designed to (1) calculate atmospheric input functions for the application of ^85^Kr as a dating tracer and (2) to investigate atmospheric circulation based on a two-box model of the atmosphere. Different functions were implemented, to (1) filter the data, (2) fit polynomials and running means, (3) extrapolate fits from the northern to the southern hemisphere, (4) calculate interhemispheric exchange times and ^85^Kr emission rates and (5) export data to a csv file.

Although the model is designed to evaluate atmospheric ^85^Kr datasets, some functionality and basic concepts can be applied to other dating tracers, like tritium and SF_6_.

• Standardized method to systematically analyse atmospheric ^85^Kr activity concentration time series for dating water and ice and to investigate atmospheric circulation.
• Easily modifiable python script to adapt functions for similar data analysis procedures.

## Graphical abstract

**Figure:**
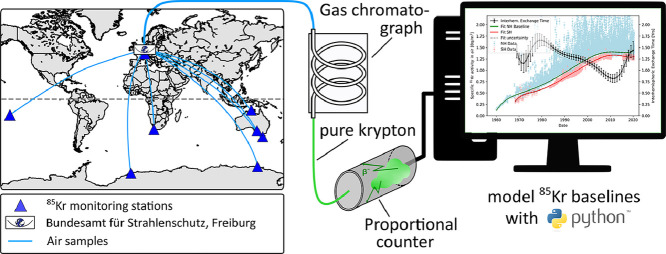

Specifications Table

**Table:** 

Subject Area	Environmental Science
More specific subject area	Data analysis
Method name	Analysis of atmospheric ^85^Kr data collection
Name and reference of original method	Not applicable
Resource availability	To reproduce the fits and models, the data collection published in the corresponding Data in Brief paper is required [1].

## Method details

With its ideal geochemical and geophysical properties as a noble gas, ^85^Kr is a valuable tracer for various applications in Earth sciences [2,3]. For dating groundwater, oceanwater and ice with ^85^Kr, a good knowledge of the atmospheric concentration during the infiltration or formation process of the investigated water or ice body is crucial. Over the past 60 years, the origin of ^85^Kr in the atmosphere can be considered purely anthropogenic, as its main source are nuclear reprocessing plants. The activity and location of nuclear reprocessing plants varied strongly over this time period, resulting in large temporal and regional changes of atmospheric ^85^Kr concentrations [4]. These trends and fluctuations are clearly detectable in the ^85^Kr datasets at the monitoring stations, which form the basis of the modelling tool that is presented here. This data analysis and plotting tool is written in python 3.8 and is designed to evaluate a comprehensive ^85^Kr dataset [1] for dating water and ice and to investigate atmospheric circulation [5]. However, certain functions of this tool can be applied to similar datasets as well.

For a better understanding of the individual functions of the model, they are applied to the ^85^Kr data collection in [1]. The main script *functions.py* as well as an example script *example.py* together with a *requirements.txt* and a *readme.txt* file can be found in the supplementary material.

### The ^85^Kr data collection

**Fig. 1:**
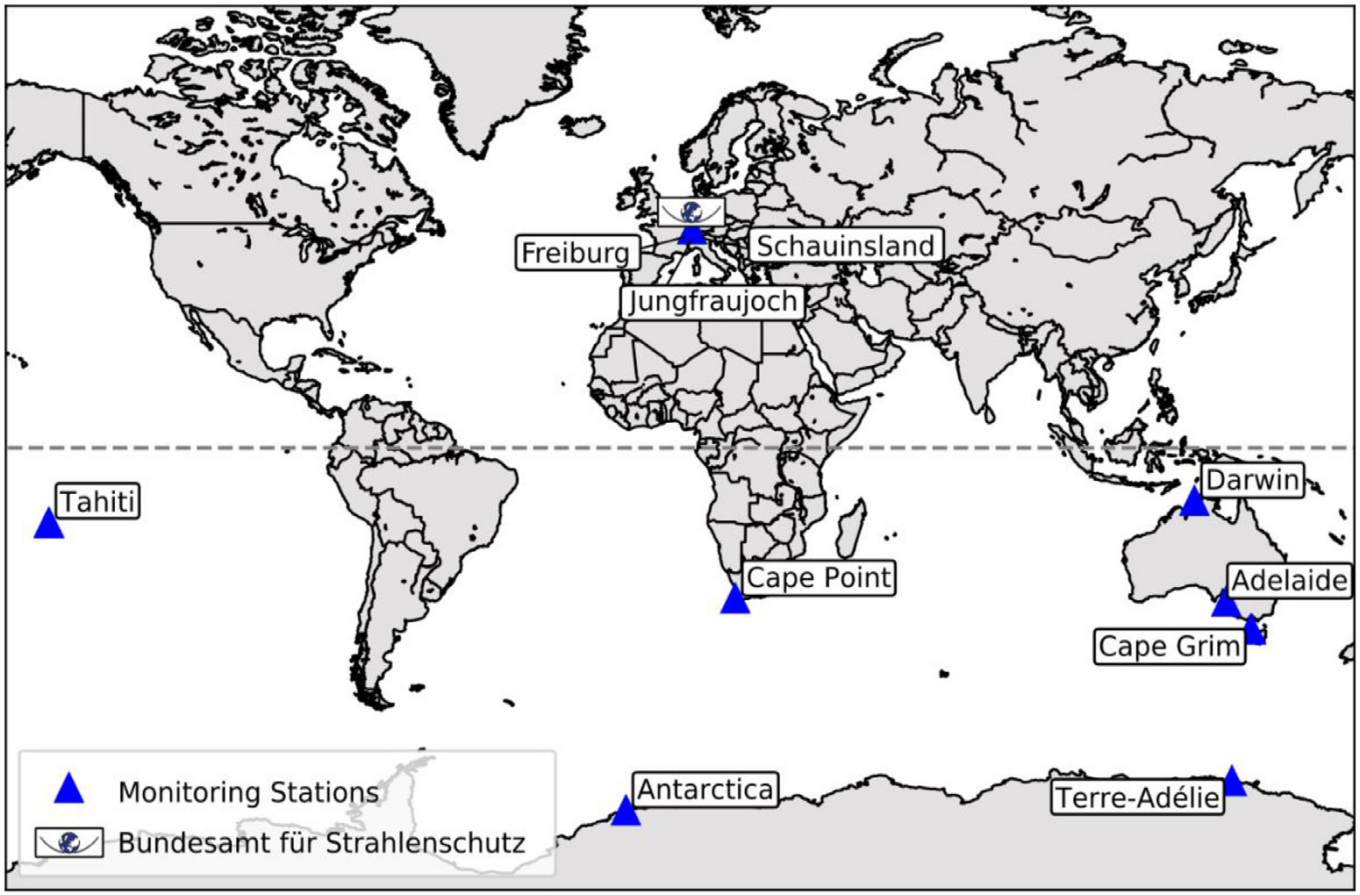
Map with all monitoring stations that contribute to the data collection the python model is based on. Furthermore, the *Bundesamt für Strahlenschutz* in Germany is shown, which processes the gathered samples and determines their ^85^Kr activity concentration.

The python model has been written for a specific ^85^Kr data collection which comprises 11 individual datasets from various monitoring stations around the globe (Fig. 1). In total, it contains about 8000 atmospheric ^85^Kr activity concentrations measured over the past 60 years. The typical sample collection and preparation process happens in two steps. First, about 5 mL of krypton from 10 m³ of air is collected over the course of one week, by trapping noble gases, including krypton, on liquid nitrogen cooled activated charcoal [6]. The krypton is then released from the charcoal trap and the enriched gas sample is sent to the laboratories of the *Bundesamt für Strahlenschutz* (Federal Office for Radiation Protection), in Freiburg, Germany. Here, the krypton fraction is separated by gas chromatography and transferred into gas proportional counters to determine the ^85^Kr activity of the sample via decay counting [7]. The measurement uncertainty of this ^85^Kr analysis method is about 3%.

### The model

**Fig. 2:**
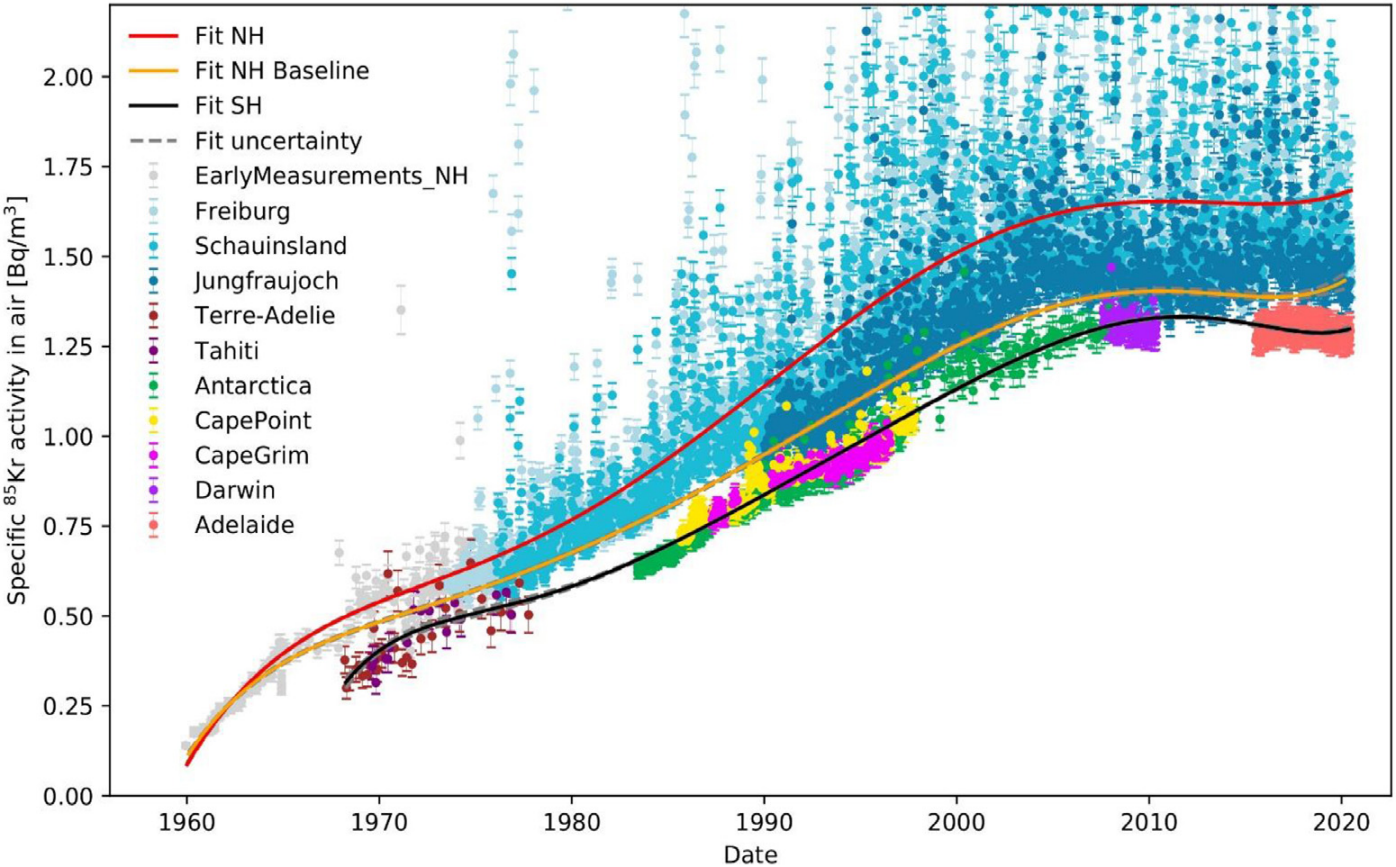
The ^85^Kr activity concentration in ground level air measured in samples from different monitoring stations are plotted against sampling date. The solid lines are polynomial fits of degree 5 (red) and 7 (orange, black) and represent local (red), northern hemispheric (orange) and southern hemispheric (black) ^85^Kr input functions. The dashed grey lines represent the errors of the fits. (For interpretation of the references to colour in this figure legend, the reader is referred to the web version of this article.)

In Fig. 2, the complete data collection is plotted. The northern hemisphere data is represented by two distinct models: First, a polynomial fit of degree 5 (red line) is used to fit the ^85^Kr data of the 3 northern hemispheric monitoring stations (Freiburg, Schauinsland, Jungfraujoch). This fit represents the average trend of ^85^Kr emissions in central Europe. Secondly, a 7th degree polynomial is fitted to the minima of the northern hemisphere data within a certain time step, representing the northern hemispheric ^85^Kr activity concentration undisturbed of local ^85^Kr sources (a baseline) and used for calculating interhemispheric exchange times. The southern hemisphere data is represented by a polynomial fit to the data of 7th degree.

In the following section, the term ‘dataset’ refers to three arrays with n entries, with the first array being the sampling date, the second array the corresponding ^85^Kr activity concentration and the third array being the measurement uncertainty of these concentrations. The number n refers to the total number of measurements in a single dataset.

The functions of the model that are used to generate the fits presented in Fig. 2 are *minDays, poly* and *errPoly*. The values for the baseline of the northern hemispheric dataset are generated with the *minDays* function, which takes a data set and the integer *days* as parameter. The given dataset is reduced to one entry for each time interval, which represents the minimum ^85^Kr activity concentration of the given period of days.

The *poly* function takes a dataset and an integer *polyDegree* as parameters and returns the polynomial fit of degree ‘*polyDegree*’ to the given dataset.

The *errPoly* function estimates the corresponding uncertainties of the polynomial fits based on Monte Carlo simulations. It takes a dataset, the corresponding integer *polyDegree* and the integer *iteration* as parameters. An *iteration* of 1000 means that 1000 synthetic datasets based on the original dataset are generated. A function of the python *numpy* module is used, which generates a synthetic data point for each real data point based on a gaussian distribution around the measured ^85^Kr activity concentration with the error of the measurement as the standard deviation. A polynomial fit of degree *polyDegree* is then fitted to each synthetic dataset resulting in 1000 possible fitted values for each point in time. The standard deviation of these 1000 data points is calculated and taken as the error of the fit for this specific time.

The fit (black line) shown in Fig. 2 represents a polynomial fit of degree 7 to all southern hemispheric datasets. The dashed grey line shortly above and below is the error of that fit generated by adding or subtracting the standard deviation calculated by the *errPoly* function.

The orange fit represents the baseline of the northern hemispheric datasets. It was generated by first filtering the northern hemispheric datasets with the *minDays* function with the parameter ‘*days’* set to 90. Then a polynomial fit of degree 7 (*poly*) as well as the error of that fit (*errPoly*) was fitted to the resulting dataset. The red line as well as the corresponding dashed grey line was generated by applying the poly and the *errPoly* function to all northern hemispheric data. For illustrative purposes, a *polyDegree* of 5 is taken here, resulting in a smoother fit.

**Fig. 3:**
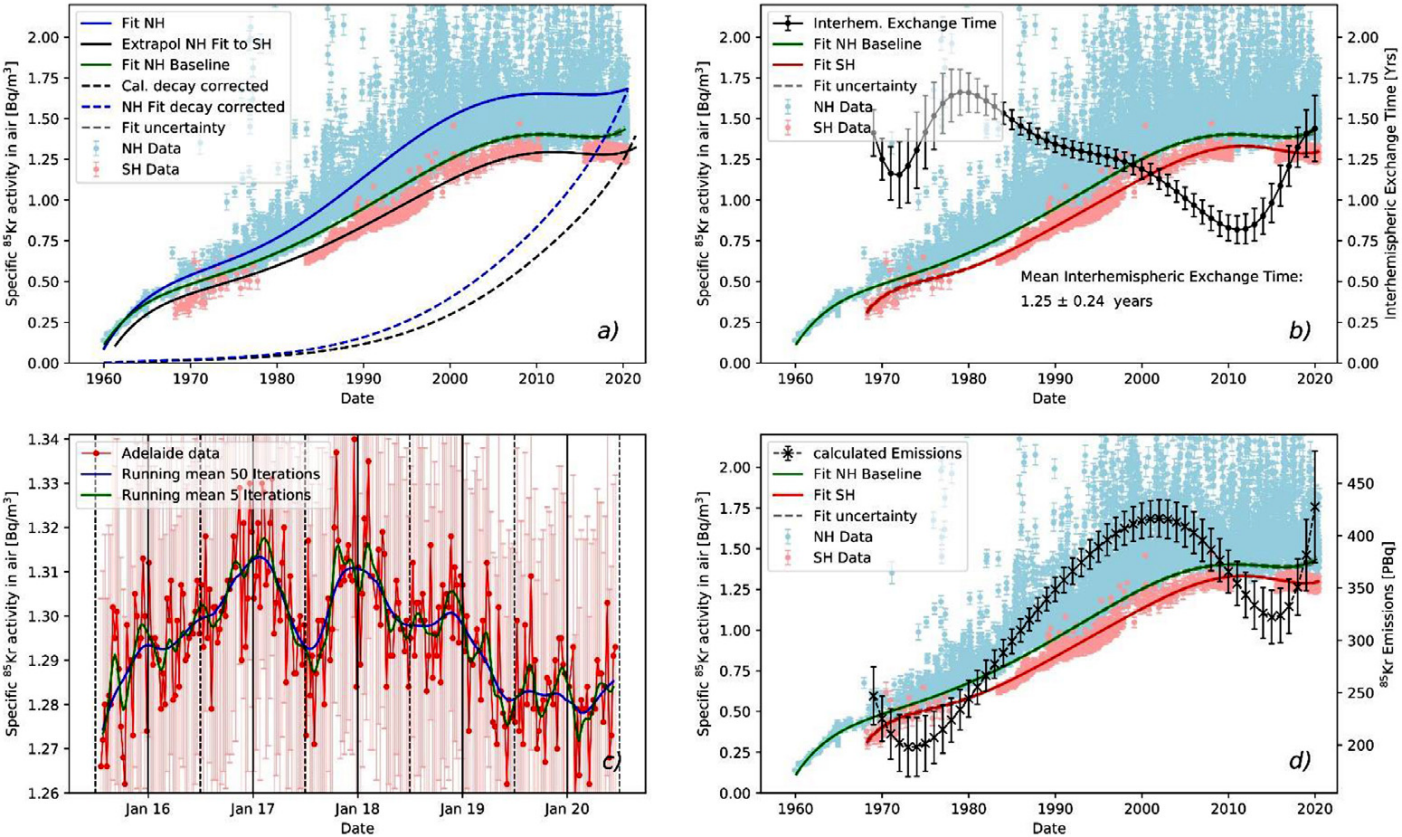
The different plots represent different features of the model: a) the northern hemispheric baseline (green line) is extrapolated to the southern hemisphere (solid black line) with the *extrapolate* function. The dashed lines are the decay corrected ^85^Kr input functions of the local northern hemispheric fit (solid blue line) and of the extrapolated southern hemisphere fit, created with the *decay* function. b) The interhemispheric exchange time is calculated on an annual basis from the fit to the southern hemisphere data and the northern hemispheric baseline with the *interhem* function. c) With the *runningMean* function of 5 and 50 iterations, a running mean is plotted to the Adelaide data to smooth out short term variability of the data. d) The ^85^Kr emission rate is calculated on an annual basis from the fit to the southern hemispheric data and the northern hemispheric baseline with the *emissions* function. (For interpretation of the references to colour in this figure legend, the reader is referred to the web version of this article.)

The three fits displayed in Fig. 2 represent atmospheric ^85^Kr input functions which are essential for the application of ^85^Kr as a dating tracer for water and ice. More details and further implications of the fits shown in Figs. 2 and 3 are discussed in the original research paper [5].

The main sources of atmospheric ^85^Kr are nuclear reprocessing plants, which are mostly located in the northern hemisphere. Therefore, many features of the python script are based on a simple atmospheric two box model, in which the northern and the southern hemispheres are treated as two well mixed boxes with specific ^85^Kr activity concentrations $C^{NH}$ and $C^{SH}$, time independent air mass flux across the equator (1/$\tau_{ex}$) and a source term $S^{NH}$ applied only to the northern hemisphere. With the decay constant λ of ^85^Kr, the differential equations that govern the hemispheric ^85^Kr activity concentrations can be written as [8]:

(1) $$\frac{dC^{NH}}{dt}=-\lambda \cdot C^{NH}-\left(\frac{C^{NH}-C^{SH}}{\tau_{ex}}\right)+S^{NH}$$

(2) $$\frac{dC^{SH}}{dt}=-\lambda \cdot C^{SH}+\left(\frac{C^{NH}-C^{SH}}{\tau_{ex}}\right)$$

The interhemispheric exchange time can then be calculated as:

(3) $$\tau_{ex}=\left(\frac{C^{NH}-C^{SH}}{\frac{dC^{SH}}{dt}+\lambda \cdot C^{SH}}\right)$$

Furthermore, the source term is given by:

(4) $$S^{NH}=\;\frac{dC^{NH}}{dt}+\frac{dC^{SH}}{dt}+\lambda \cdot \left(C^{NH}+C^{SH}\right)$$

When only the trend of the northern hemispheric ^85^Kr activity concentration is known and an interhemispheric exchange time is given, the southern hemispheric concentration can be modelled:

(5) $$C^{SH}\left(t\right)=C^{NH}\left(t-\tau_{ex}\right)\cdot \exp\left(-\tau_{ex}\cdot \lambda \right)$$

To better analyse short term trends within the data, a running mean can be calculated. This is done with the *runningMean* function, which takes a dataset and the integer *runningMeanCycles* as parameters. For each cycle, the input data is smoothened by a 3rd order low pass filter of the form:

(6) $$C_{n}^{Av}=\frac{1}{3}C_{n-1}+\frac{1}{3}C_{n}+\frac{1}{3}C_{n+1}$$

In Fig. 3a two features of the model are presented. First, the black line represents the extrapolation of the northern hemispheric baseline (green line) to the southern hemisphere according to Eq. (5). This is done with the *extrapolate* function which takes a dataset and a float value for the interhemispheric exchange time in years (here 1.25) as input and returns the extrapolated dataset. The dashed lines in the figure are the decay corrected activity concentrations relative to a given date (here 1st of August 2020). The corresponding python function is the *decay* function, which takes a dataset and the reference date as input. The decay constant is set to 15.5 years for ^85^Kr and can easily be replaced to apply this functionality to other radioactive tracers, like tritium.

The interhemispheric exchange time (Fig. 3b) is estimated according to Eq. (3). The required function for this procedure is the *interhem* function, which takes the polynomial fit to the southern hemispheric datasets, as well as the polynomial fit to the baseline of the northern hemispheric datasets including their fitting errors as parameters. It then calculates the interhemispheric exchange time on an annual basis and returns the three arrays date, interhemispheric exchange time and error of the interhemispheric exchange time. The error is calculated according to the Gaussian error propagation from the uncertainties of the fits.

The trends within the Adelaide dataset are analysed in Fig. 3c by fitting 5 and 50 iterations of the running mean to the original data, according to Eq. (6).

The source term is calculated on an annual basis (Fig. 3d). The function *emissions* is written analogously to the *interhem* function and is based on Eq. (4).

Two additional functions of the python model are not represented in the figures. The *gofNH_SH* function calculates how well the extrapolated northern hemispheric fit describes the southern hemispheric data. This is done by calculating the χ² value of the extrapolated fit. The *export* function writes the modelled dates, values and errors of a polynomial fit to a specific dataset to a csv file.

## Declaration of Competing Interest

The authors declare that they have no known competing financial interests or personal relationships that could have appeared to influence the work reported in this paper.

## Appendix Supplementary materials

Image, application 1

Image, application 2

Image, application 3

Image, application 4
